# Supplementary material for: Molecular Characterization of Seasonal Influenza A and B from Hospitalized Patients in Thailand in 2018–2019
Source: Viruses. 2021 May 25;13(6):977. doi: 10.3390/v13060977 (PMC8228477; doi:10.3390/v13060977)
Supplement: Supplementary file 1 [file viruses-13-00977-s001.zip › viruses-1127845-supplementary.pdf]

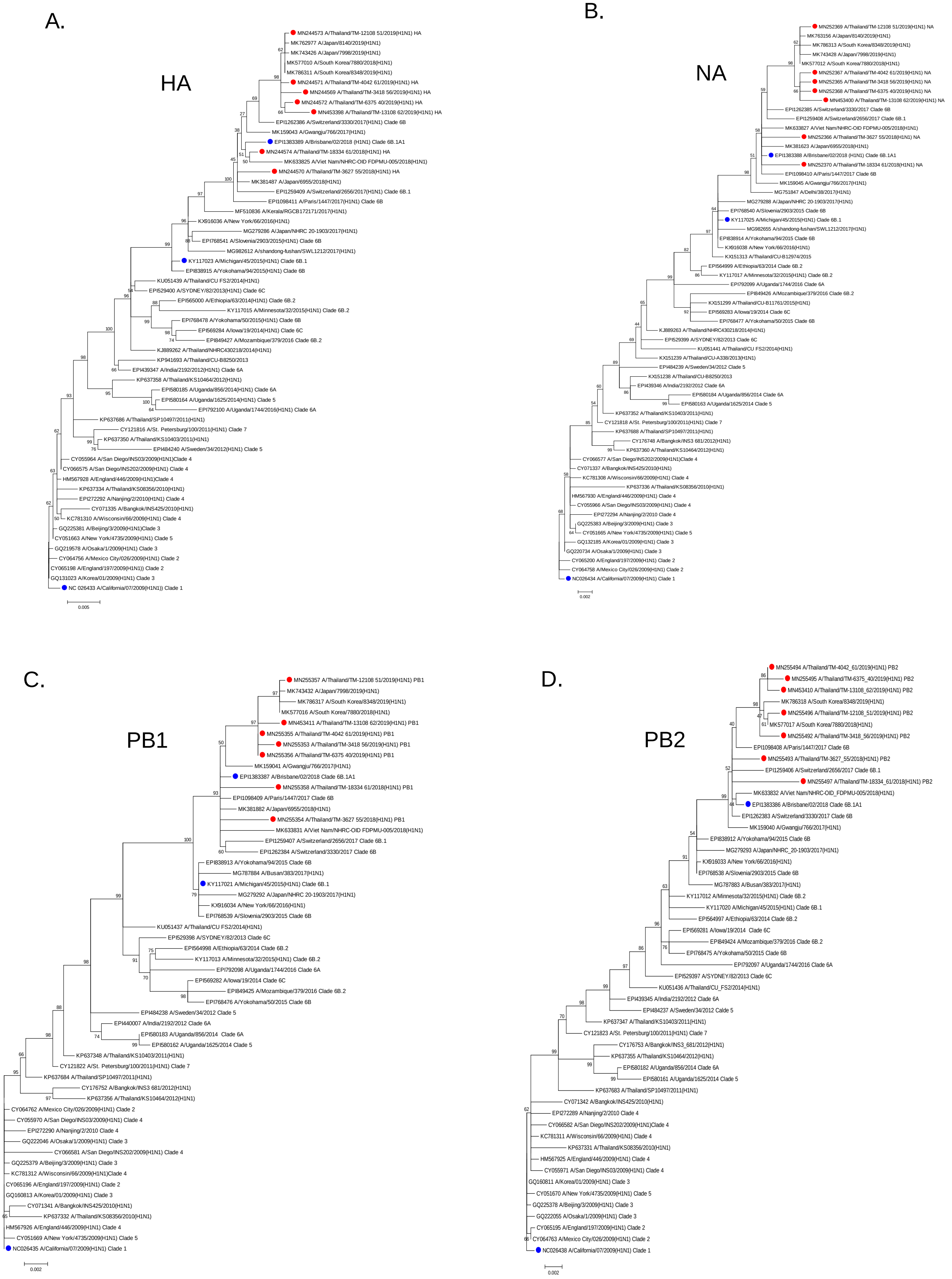

Supplementary Figure 1. Phylogenetic tree of HA (A), NA (B), PB1 (C), and PB2 (D) genes of influenza A/H1N1 Thai strains was inferred by using the Maximum Likelihood method based on the Tamura 3-parameter model. Red circles indicate A/H1N1 current isolates and blue circles indicate A/H1N1 vaccine strains.

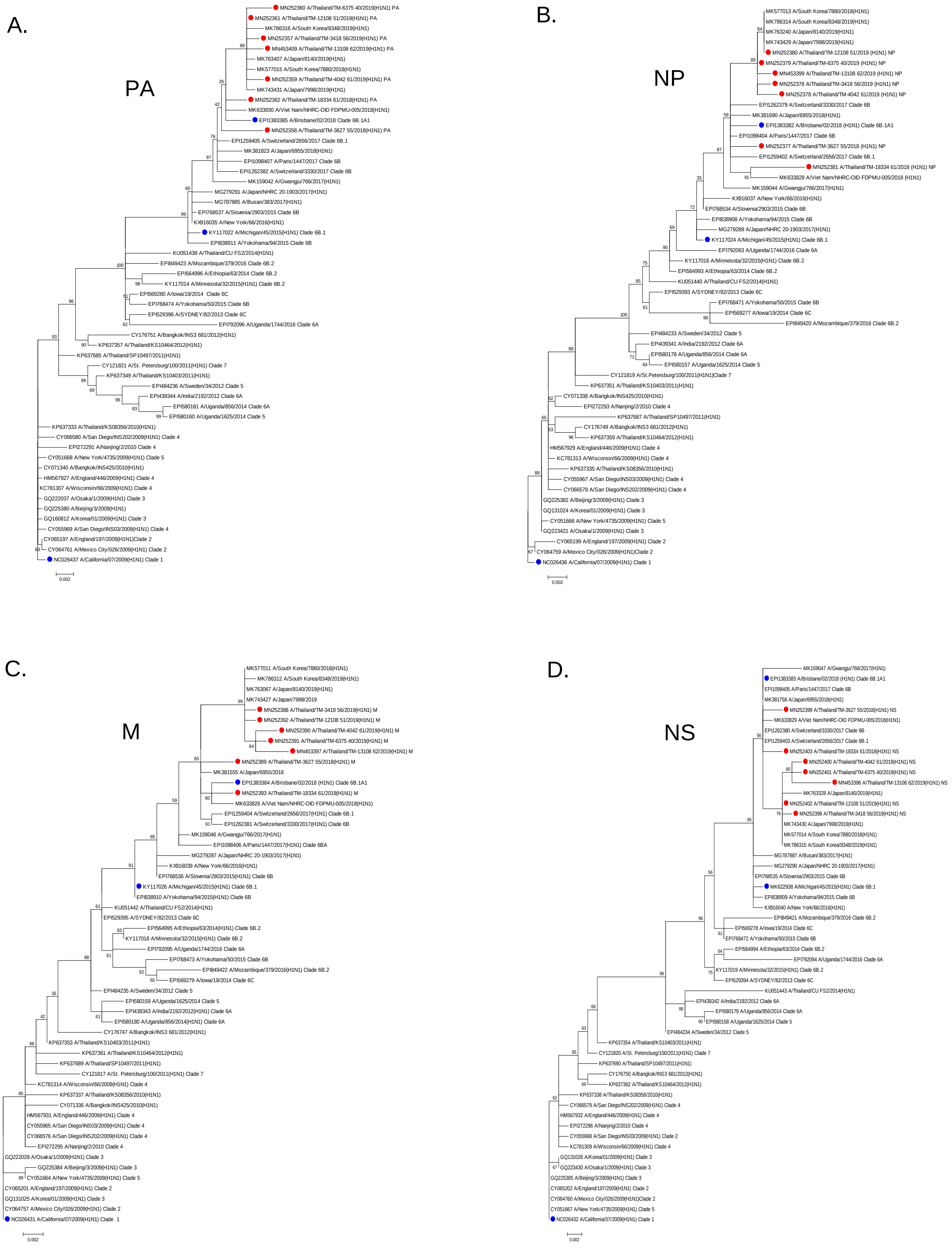

Supplementary Figure 2. Phylogenetic tree of PA (A), NP (B), M (C), and NS (D) genes of influenza A/H1N1 strains was inferred by using the Maximum Likelihood method based on the Tamura 3-parameter model. Red circles indicate A/H1N1 current isolates and blue triangles indicate A/H1N1 vaccine strains.

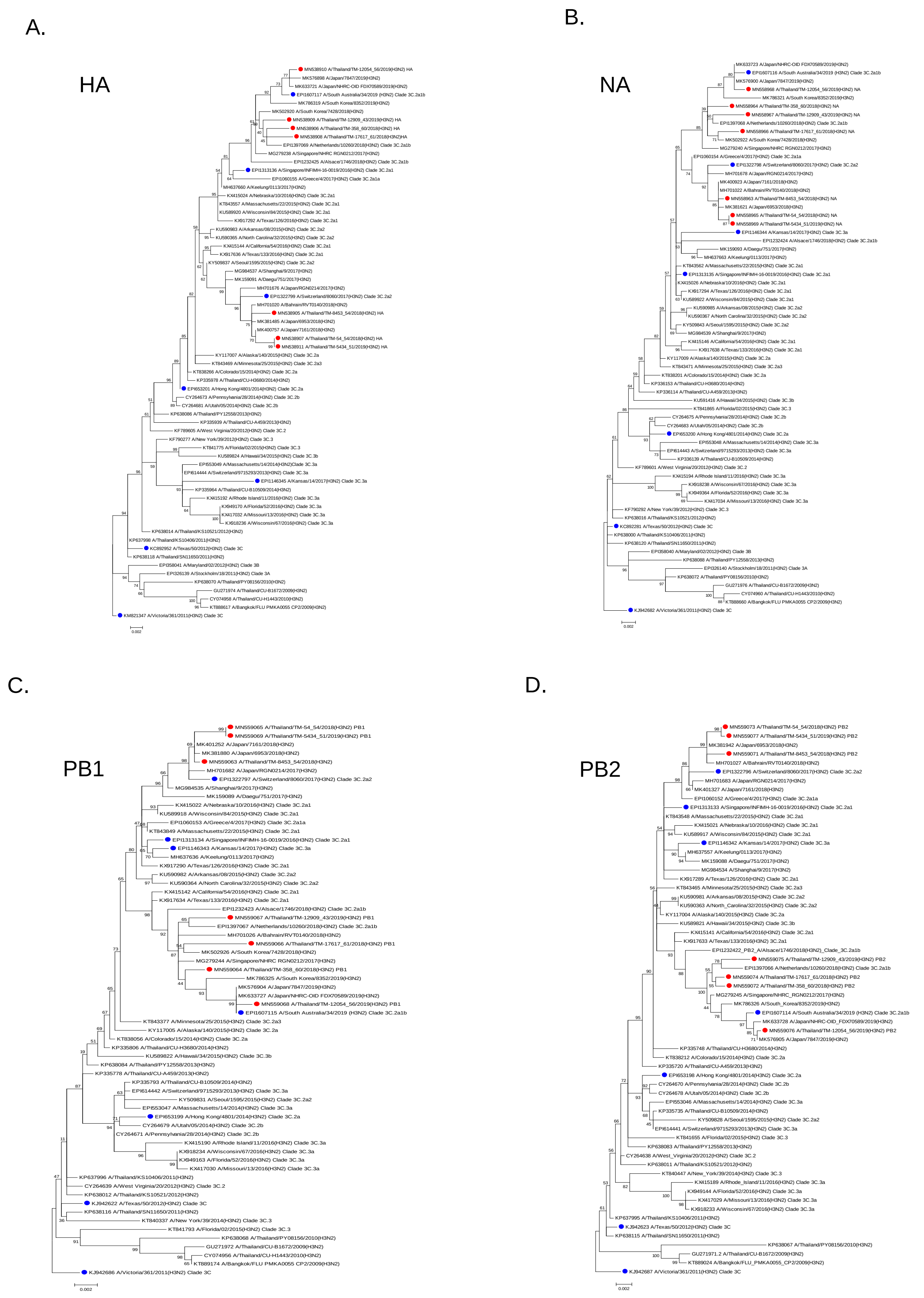

Supplementary Figure 3. Phylogenetic tree of HA (A), NA (B), PB1 (C), and PB2 (D) genes of influenza A/H1N1 Thai strains was inferred by using the Maximum Likelihood method based on the Tamura 3-parameter model. Red circles indicate A/H1N1 current isolates and blue triangles indicate A/H1N1 vaccine strains.

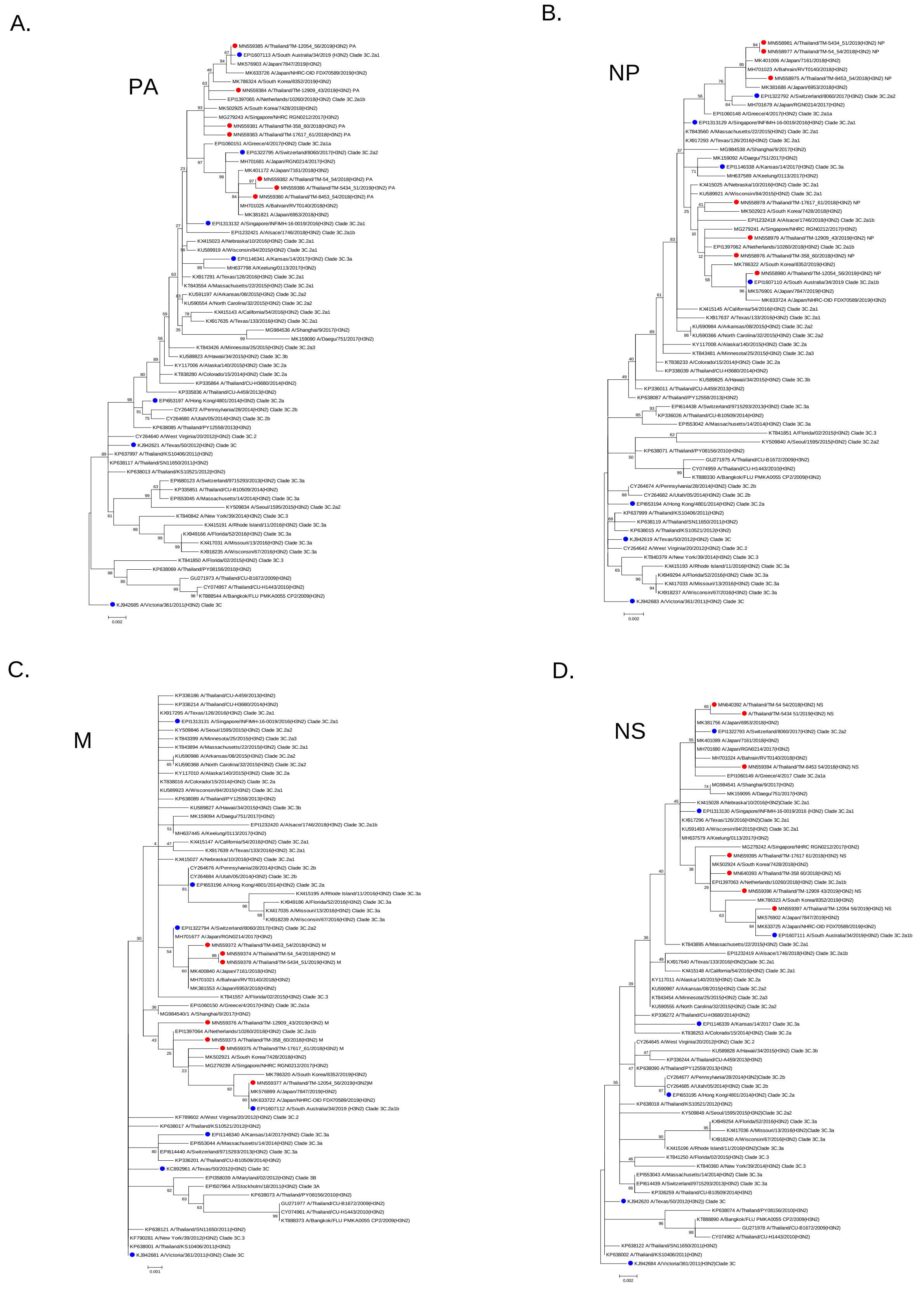

Supplementary Figure 4. Phylogenetic tree of PA (A), NP (B), M (C), and NS (D) genes of influenza A/H3N2 Thai strains was inferred by using the Maximum Likelihood method based on the Tamura 3-parameter model. Red circles indicate A/H3N2 current isolates and blue triangles indicate A/H3N2 vaccine strains.

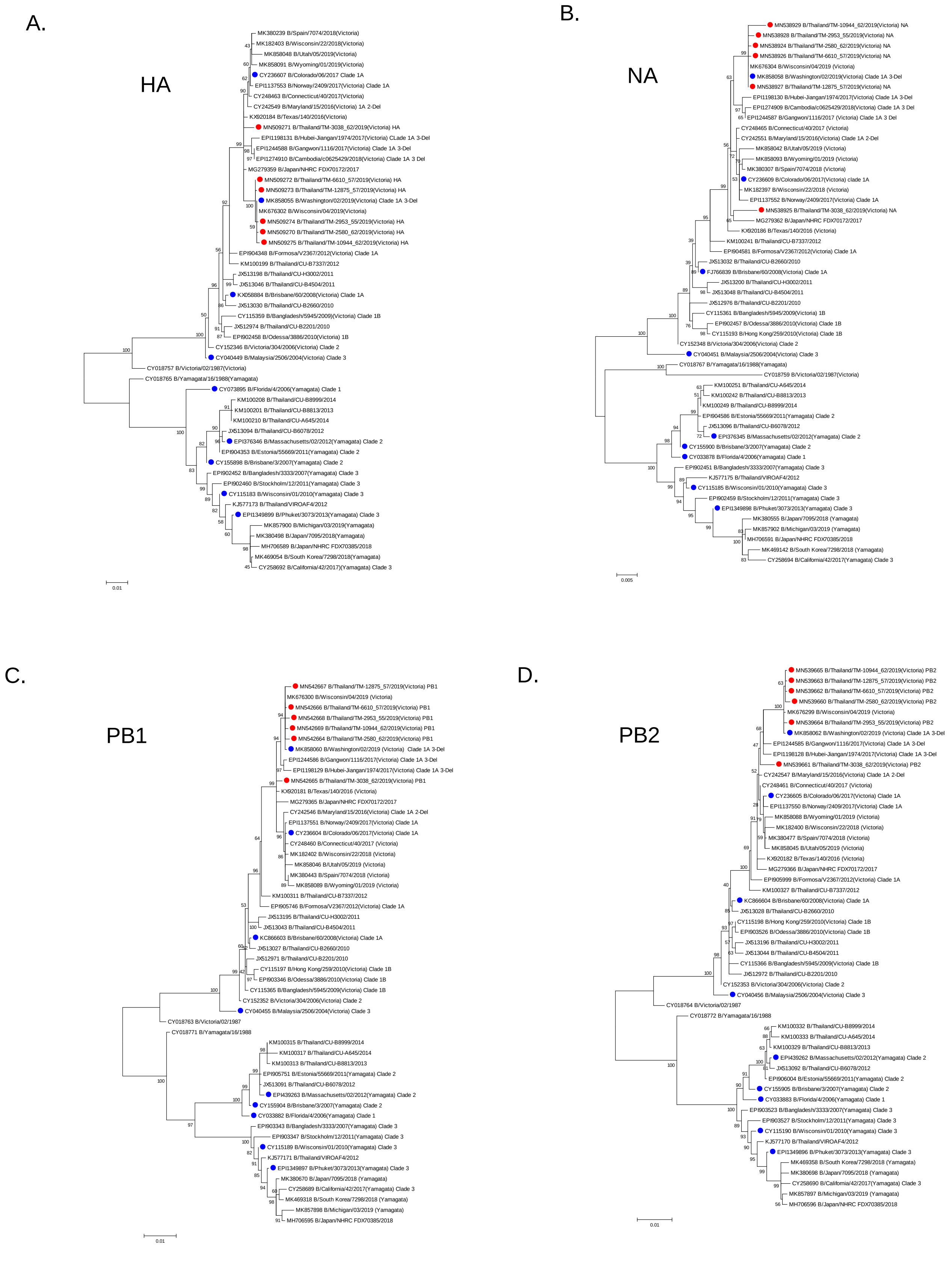

Supplementary Figure 5. Phylogenetic tree of HA (A), NA (B), PB1 (C), and PB2 (D) genes of influenza B/Vic Thai strains was inferred by using the Maximum Likelihood method based on the Tamura 3-parameter model. Red circles indicate B/Victoria current isolates and blue circles indicate influenza B vaccine strains.

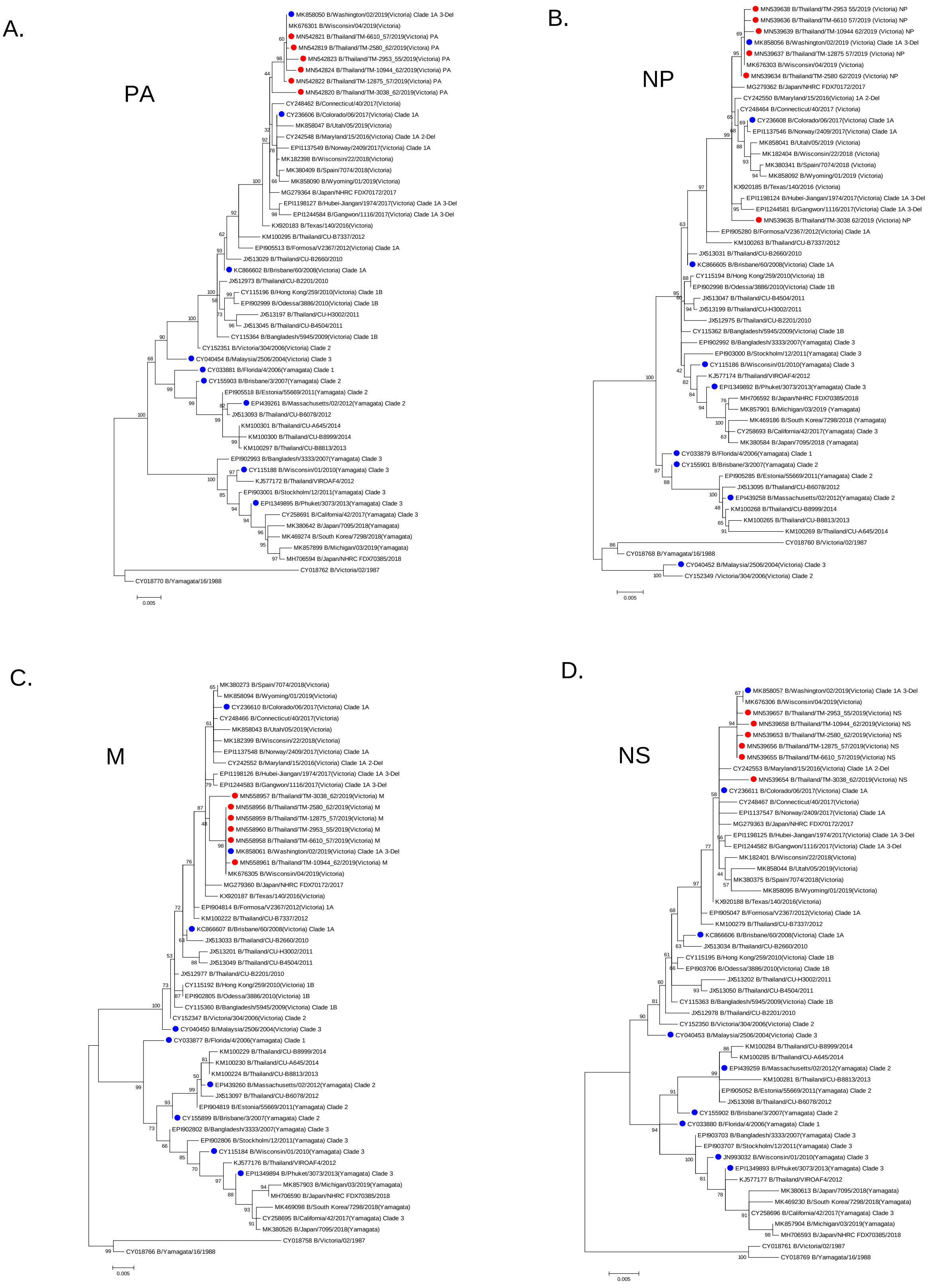

Supplementary Figure 6. Phylogenetic tree of PA (A), NP (B), M (C), and NS (D) genes of influenza B/Victoria Thai strains was inferred by using the Maximum Likelihood method based on the Tamura 3-parameter model. Red circles indicate B/Victoria current isolates and blue circles indicate influenza B vaccine strains.

Supplementary Table 1. List of identification (ID) numbers, strain names, collection dates, and GenBank accession numbers of influenza virus isolates from hospitalized patients at

No.	Isolate ID	Strain name	Collection date	GenBank ID Segment 1	GenBank ID Segment 2	GenBank ID Segment 3	GenBank ID Segment 4	GenBank ID Segment 5	GenBank ID Segment 6	GenBank ID Segment 7	GenBank ID Segment 8
1	TM-3418_56	A/Thailand/TM-3418_56/2019(H1N1)	26-03-2019	MN255492	MN255353	MN252357	MN244569	MN252376	MN252365	MN252388	MN252398
2	TM-3627_55	A/Thailand/TM-3627_55/2018(H1N1)	08-08-2018	MN255493	MN255354	MN252358	MN244570	MN252377	MN252366	MN252389	MN252399
3	TM-4042_61	A/Thailand/TM-4042_61/2019(H1N1)	15-02-2019	MN255494	MN255355	MN252359	MN244571	MN252378	MN252367	MN252390	MN252400
4	TM-6375_40	A/Thailand/TM-6375_40/2019(H1N1)	21-02-2019	MN255495	MN255356	MN252360	MN244572	MN252379	MN252368	MN252391	MN252401
5	TM-12108_51	A/Thailand/TM-12108_51/2019(H1N1)	03-02-2019	MN255496	MN255357	MN252361	MN244573	MN252380	MN252369	MN252392	MN252402
6	TM-18334_61	A/Thailand/TM-18334_61/2018(H1N1)	22-09-2018	MN255497	MN255358	MN252362	MN244574	MN252381	MN252370	MN252393	MN252403
7	TM-13108_62	A/Thailand/TM-13108_62/2018(H1N1)	28-06-2019	MN453410	MN453411	MN453409	MN453398	MN453399	MN453400	MN453397	MN453396
8	TM-2580_62	B/Thailand/TM-2580_62/2019	03-02-2019	MN539660	MN542664	MN542819	MN509270	MN539634	MN538924	MN558956	MN539653
9	TM-3038_62	B/Thailand/TM-3038_62/2019	11-02-2019	MN539661	MN542665	MN542820	MN509271	MN539635	MN538925	MN558957	MN539654
10	TM-6610_57	B/Thailand/TM-6610_57/2019	05-03-2019	MN539662	MN542666	MN542821	MN509272	MN539636	MN538926	MN558958	MN539655
11	TM-12875_57	B/Thailand/TM-12875_57/2019	17-04-2019	MN539663	MN542667	MN542822	MN509273	MN539637	MN538927	MN558959	MN539656
12	TM-2953_55	B/Thailand/TM-2953_55/2019	21-05-2019	MN539664	MN542668	MN542823	MN509274	MN539638	MN538928	MN558960	MN539657
13	TM-10944_62	B/Thailand/TM-10944_62/2019	01-06-2019	MN539665	MN542669	MN542824	MN509275	MN539639	MN538929	MN558961	MN539658
14	TM-8453_54	A/Thailand/TM-8453_54/2018(H3N2)	10-08-2018	MN559071	MN559063	MN559380	MN538905	MN558975	MN558963	MN559372	MN559394
15	TM-358_60	A/Thailand/TM-358_60/2018(H3N2)	02-08-2018	MN559072	MN559064	MN559381	MN538906	MN558976	MN558964	MN559373	MN640393
16	TM-54_54	A/Thailand/TM-54_54/2018(H3N2)	11-09-2018	MN559073	MN559065	MN559382	MN538907	MN558977	MN558965	MN559374	MN640392
17	TM-17617_61	A/Thailand/TM-17617_61/2018(H3N2)	13-09-2018	MN559074	MN559066	MN559383	MN538908	MN558978	MN558966	MN559375	MN559395
18	TM-12909_43	A/Thailand/TM-12909_43/2019(H3N2)	17-01-2019	MN559075	MN559067	MN559384	MN538909	MN558979	MN558967	MN559376	MN559396
19	TM-12054_56	A/Thailand/TM-12054_56/2019(H3N2)	18-03-2019	MN559076	MN559068	MN559385	MN538910	MN558980	MN558968	MN559377	MN559397
20	TM-5434_51	A/Thailand/TM-5434_51/2019(H3N2)	01-04-2019	MN559077	MN559069	MN559386	MN538911	MN558981	MN558969	MN559378	MN647629

Supplementary Table 2. Hemagglutination Inhibition titers

Viruses	Subtypes	HI Titer
A/Thailand/TM-18334_61/2018	H1N1	64-1024
A/Thailand/TM-3627_55/2018	H1N1	128-512
A/Thailand/TM-12108_51/2019	H1N1	64-128
A/Thailand/TM-6375_40/2019	H1N1	64-128
A/Thailand/TM-4042_61/2019	H1N1	32-128
A/Thailand/TM-3418_56/2019	H1N1	64-128
A/Thailand/TM-13108_62/2018	H1N1	16-256
B/Thailand/TM-6610_57/2019	B/Vic	16-128
B/Thailand/TM-3038_62/2019	B/Vic	32-128
B/Thailand/TM-12875_57/2019	B/Vic	32-128
B/Thailand/TM-2580_62/2019	B/Vic	32-64
B/Thailand/TM-2953_55/2019	B/Vic	64-128
B/Thailand/TM-10944_62/2019	B/Vic	32-128
A/Thailand/TM-8453_54/2018	H3N2	128-256
A/Thailand/TM-17617_61/2018	H3N2	16-64
A/Thailand/TM-12054_56/2019	H3N2	32-64
A/Thailand/TM-12909_43/2019	H3N2	32-64
A/Thailand/TM-358_60/2018	H3N2	32
A/Thailand/TM-54_54/2018	H3N2	32
A/Thailand/TM-5434_51/2019	H3N2	8-16

Supplementary Table 3. Amino acid substitutions of NA and M of Thai A/H1N1 strains.

Representative Strain	Clade	Amino acid signature at a given residue*														
		NA								M						
		51	74	77	81	188	389	416	462	80	192	208	230	261	280	308
A/Brisbane/02/2018	6B.1A1	Q	F	R	A	T	I	D	T	I	V	K	R	H	H	A
A/Michigan/45/2015	6B.1	•	•	G	V	I	•	•	•	•	•	•	•	•	•	•
A/California/07/2009	1	•	•	G	V	I	•	•	•	V	M	Q	K	Y	Y	T
A/Thailand/TM-3418_56/2019	6B.1	K	S	•	•	•	K	N	I	•	•	•	•	•	•	•
A/Thailand/TM-3627_55/2018	6B.1A1	•	•	•	•	•	•	•	•	•	•	•	•	•	•	•
A/Thailand/TM-4042_61/2019	6B.1	K	S	•	•	•	K	N	I	•	M	•	•	•	•	•
A/Thailand/TM-6375_40/2019	6B.1	K	S	•	•	•	K	N	I	•	•	•	•	•	•	•
A/Thailand/TM-12108_51/2019	6B.1	K	S	•	•	•	•	N	I	•	•	•	•	•	•	•
A/Thailand/TM-13108_62/2019	6B.1	K	S	•	•	•	K	N	I	•	•	•	•	•	•	•
A/Thailand/TM-18334_61/2018	6B.1A1	•	•	•	•	•	•	D	•	•	•	•	•	•	•	•

* Amino acid signatures are compared to B/Brisbane/02/2018, A/Michigan/45/2015, and A/California/07/2009

Supplementary Table 4. Amino acid substitutions of NP, NS, PB1, PB2, PA of Thai A/H1N1 strains.

Representative Strain	Clade	Amino acid signature at a given residue*														
		NP				NS				PB1		PB2			PA	
		106	111	128	431	55	80	155	242	200	386	225	453	667	61	100
A/Brisbane/02/2018	6B.1A1	I	T	Q	V	K	T	A	V	K	G	T	V	T	I	T
A/Michigan/45/2015	6B1	•	•	•	•	•	•	•	•	•	•	P	•	I	•	A
A/California/07/2009	1	V	M	L	•	E	•	•	•	•		S	•	I	V	A
A/Thailand/TM-3418_56/2019	6B1	•	•	•	I	•	A	T	I	R	S	•	I	I	•	•
A/Thailand/TM-3627_55/2018	6B.1A1	•	•	•	•	•	•	•	•	•	•	•	•	I	•	•
A/Thailand/TM-4042_61/2019	6B1	•	•	•	I	•	A	T	I	R	S	•	I	I	•	•
A/Thailand/TM-6375_40/2019	6B1	•	•	•	I	•	A	T	I	R	S	•	I	I	•	•
A/Thailand/TM-12108_51/2019	6B1	•	•	•	I	•	A	T	I	R	S	•	I	I	•	•
A/Thailand/TM-13108_62/2019	6B1	•	•	•	I	•	A	T	I	R	S	•	I	I	•	•
A/Thailand/TM-18334_61/2018	6B.1A1	•	•	•	•	•	•	•	•	•	•	•	•	I	•	•

* Amino acid signatures are compared to B/Brisbane/02/2018, A/Michigan/45/2015, and A/California/07/2009

Supplementary Table 5. Amino acid substitutions of NA, M, NP, PA, and PB1 of Thai H3N2 strains.

Representative Strain	Clade	Amino acid signature at a given residue*														
		NA							M		NP		PA		PB1	
		79	126	176	220	303	329	386	275	306	197	418	158	222	517	586
A/South Australia/34/2019	3C.2a1b	P	L	I	N	I	S	P	P	P	I	I	R	N	I	R
A/Kansas/14/2017	3C.3a	•	P	•	K	V	T	•	S	S	V	L	K	•	•	•
A/Switzerland/8060/2017	3C.2a2	•	P	M	K	V	•	S	S	S	•	L	K	•	•	•
A/Singapore/INFIMH-16-0019/2016	3C.2a1	•	P	•	K	V	N	•	S	S	V	L	K	•	•	•
A/Thailand/TM-54_54/2018	3C.2a2	L	P	M	K	V	•	S	S	S	•	L	K	H	V	K
A/Thailand/TM-358_60/2018	3C.2a1b	•	•	•	•	•	•	•	S	•	•	L	•	•	•	•
A/Thailand/TM-5434_51/2019	3C.2a2	L	P	M	K	V	•	S	S	S	•	L	K	H	V	K
A/Thailand/TM-8453_54/2018	3C.2a2	•	P	M	K	V	•	S	S	S	•	L	K	•	•	•
A/Thailand/TM-10254_56/2019	3C.2a1b	•	•	•	•	•	•	•	•	•	•	•	•	•	•	•
A/Thailand/TM-12909_43/2019	3C.2a1b	•	•	•	•	•	•	•	S	S	•	L	•	•	•	•
A/Thailand/TM-17617_61/2018	3C.2a1b	•	•	•	•	•	•	•	S	•	•	L	•	•	•	•

* Amino acid signatures are compared to A/South Australia/34/2019, A/Kansas/14/2017, A/Switzerland/8060/2017, and A/Singapore/INFIMH-16-0019/2016.

Supplementary Table 6. Amino acid substitutions of PB2 and NS of Thai H3N2 strains

Representative Strain	Clade	Amino acid signature at a given residue*					
		PB2				NS	
		64	107	299	340	56	71
A/South Australia/34/2019	3C.2a1b	T	N	R	R	S	G
A/Kansas/14/2017	3C.3a	•	S	K	K	A	E
A/Switzerland/8060/2017	3C.2a2	•	S	K	K	•	•
A/Singapore/INFIMH-16-0019/2016	3C.2a1	•	S	K	K	•	•
A/Thailand/TM-54_54/2018	3C.2a2	I	S	K	K	•	•
A/Thailand/TM-358_60/2018	3C.2a1b	•	•	•	•	•	•
A/Thailand/TM-5434_51/2019	3C.2a2	I	S	K	K	•	•
A/Thailand/TM-8453_54/2018	3C.2a2	I	S	K	K	•	•
A/Thailand/TM-10254_56/2019	3C.2a1b	•	•	•	•	•	•
A/Thailand/TM-12909_43/2019	3C.2a1b	•	•	•	•	•	•
A/Thailand/TM-17617_61/2018	3C.2a1b	•	•	•	•	•	•

*Amino acid signatures are compared to A/South Australia/34/2019, A/Kansas/14/2017, A/Switzerland/8060/2017, and A/Singapore/INFIMH-16-0019/2016

Supplementary Table 7. Amino acid substitutions of NA, M, NP, PA, and PB1 of Thai B/Victoria strains.

Representative Strain	Clade	Amino acid signature at a given residue*												
		NA				M		NP			PA	PB1		
		99	123	139	146	285	299	9	66	453	352	208	454	474
B/Washington/02/2019	1A(△3)	S	S	C	*	I	T	M	A	A	A	V	N	I
B/Colorado/06/2017	1A(△2)	P	C	S	Q	T	P	•	•	V	•	I	D	•
B/Brisbane/60/2008	1A	P	C	S	Q	T	•	I	T	V	T	•	D	V
B/Thailand/TM-2580_62/2019	1A(△3)	•	•	•	*	•	•	I	•	•	•	•	D	•
B/Thailand/TM-2953_55/2019	1A(△3)	•	•	•	•	•	•	I	•	•	•	•	D	•
B/Thailand/TM-3038_62/2018	1A	P	C	S	Q	T	•	I	•	V	•	•	D	•
B/Thailand/TM-6610_57/2019	1A(△3)	•	•	•	*	•	•	I	•	•	•	•	D	•
B/Thailand/TM-10944_62/2019	1A(△3)	•	•	•	*	•	•	I	•	•	•	•	D	•
B/Thailand/TM-12875_57/2018	1A(△3)	•	•	•	*	•	•	I	•	•	•	•	D	•

‡Amino acid signatures are compared to A/Washington/02/2019, A/Colorado/06/2017, and A/Brisbane/60/2008.
* Indicates a stop codon.

Supplementary Table 8. Amino acid substitutions of PB2 and NS of Thai B/Vic Strains

Representative Strain	Clade	Amino acid signature at a given residue*				
		PB2		NS		
		56	492	107	201	284
B/Washington/02/2019	1A(△3)	S	T	M	Y	I
B/Colorado/06/2017	1A(△2)	N	•	•	•	•
B/Brisbane/60/2008	1A	N	I	K	H	T
B/Thailand/TM-2580_62/2019	1A(△3)	N	•	•	H	•
B/Thailand/TM-2953_55/2019	1A(△3)	N	•	•	•	•
B/Thailand/TM-3038_62/2018	1A	N	•	•	•	•
B/Thailand/TM-6610_57/2019	1A(△3)	N	•	•	•	•
B/Thailand/TM-10944_62/2019	1A(△3)	N	•	•	•	•
B/Thailand/TM-12875_57/2018	1A(△3)	N	•	•	•	•

‡ Amino acid signatures are compared to A/Washington/02/2019, A/Colorado/06/2017, and A/Brisbane/60/2008.
